# Supplementary figures and images for: Improving the efficiency of the Fukui trap as a capture tool for the invasive European green crab (Carcinus maenas) in Newfoundland, Canada
Source: PeerJ. 2019 Jan 29;7:e6308. doi: 10.7717/peerj.6308 (PMC6357871; doi:10.7717/peerj.6308)

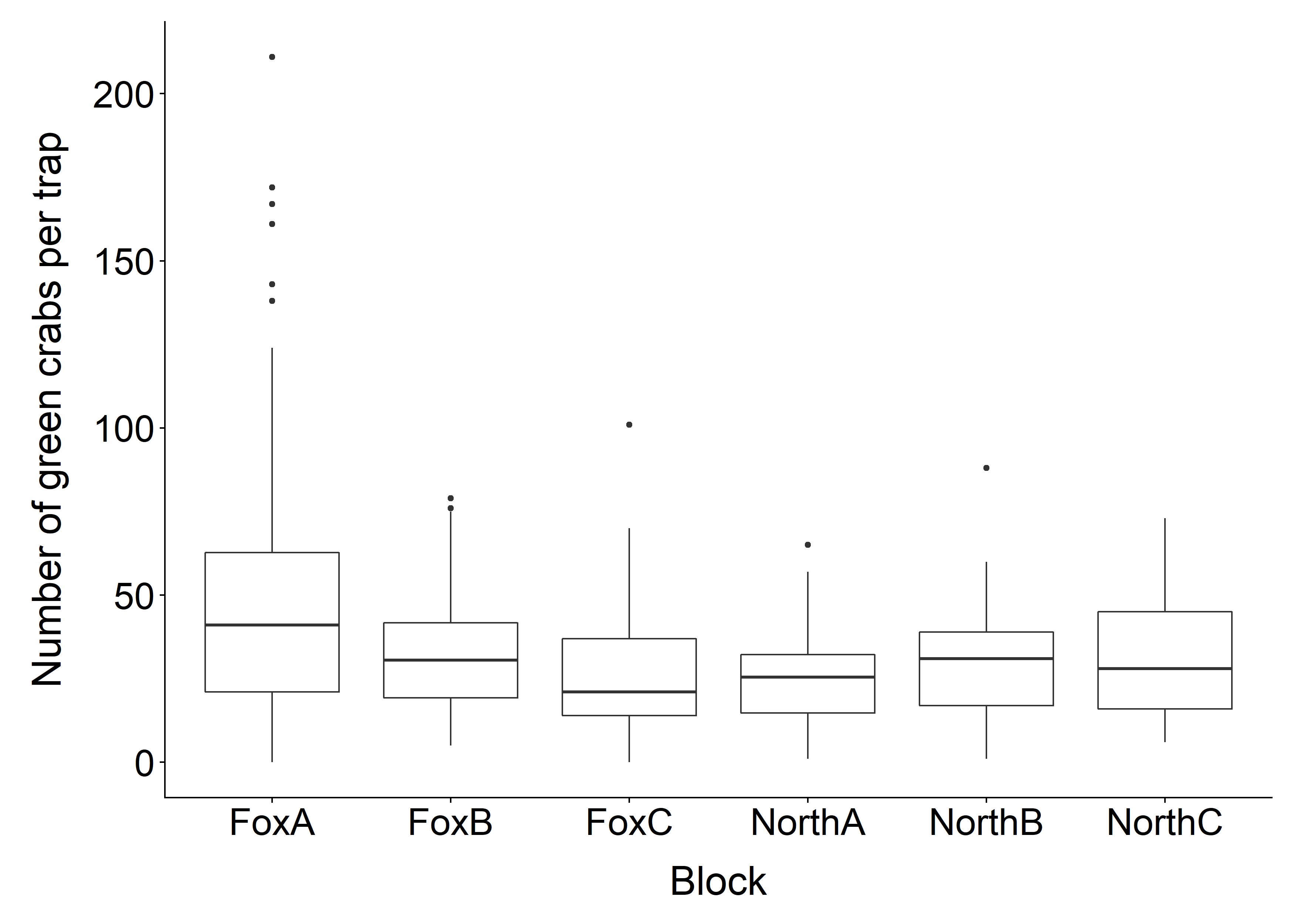

Supplement: Figure S1 — The solid black line within each box depicts the median for that trap type. The lower and upper hinges of the box correspond to the first and third quartiles, respectively. The upper whisker extends to the largest value no further than 1.5 times the inter-quartile range (1.5*IQR), and the lower whisker extends to the smallest value no further than 1.5*IQR. [file peerj-07-6308-s001.png]
